# Gentrification and Cognitive Aging: Examining Neighborhood Change and Cognitive Decline in Older Adults in the Multi-Ethnic Study of Atherosclerosis

**DOI:** 10.21203/rs.3.rs-9985996/v1

**Published:** 2026-06-18

**Authors:** Yvonne L Michael, Kari Moore, Lilah M Besser, Timothy M Hughes, Reyhaneh Akbar Nejad Yazdi, Kathleen M Hayden, Jana A Hirsch

**Affiliations:** Drexel University Dornsife School of Public Health; Drexel University Dornsife School of Public Health; University of Miami Miller School of Medicine: University of Miami School of Medicine; Wake Forest University School of Medicine; Drexel University Dornsife School of Public Health; Trinity College Dublin: The University of Dublin Trinity College; Drexel University Dornsife School of Public Health

**Keywords:** Neighborhood advantage, Gentrification, Older adult, Cognitive function, Health inequities

## Abstract

We examined whether neighborhood gentrification is associated with cognitive change among older adults in the Multi-Ethnic Study of Atherosclerosis (MESA; n = 1,731). Among participants, 23% resided in continually advantaged neighborhoods, 34% in gentrified neighborhoods, and 43% in continually disadvantaged neighborhoods (2000–2010). A Global Cognitive Composite (GCC) assessed memory, processing speed, and executive function from 2010–2024. Linear mixed-effects models with inverse probability weights estimated cognitive change over 5 years. After adjustment for confounding, GCC declined most in gentrified neighborhoods (− 0.15 SD) and least in continually advantaged neighborhoods (− 0.12 SD), with intermediate decline in continually disadvantaged neighborhoods (− 0.13 SD; p = 0.06). Neighborhood-level change may not confer cognitive protection and could carry risks, even relative to persistently disadvantaged contexts.

## 1. Introduction

Alzheimer’s disease (AD) and Alzheimer’s Disease-related dementias (ADRD) are common and increasingly prevalent in older adults.^1^ A recent multidomain lifestyle intervention provided small but statistically significant improvements in cognitive function among older adults at risk of cognitive decline and dementia, supporting the importance of identifying and addressing modifiable behavioral risk factors. Broader societal and environmental determinants may also be modifiable and influence cognition directly or through behaviors and other related processes.^2^

**Figure 1:**
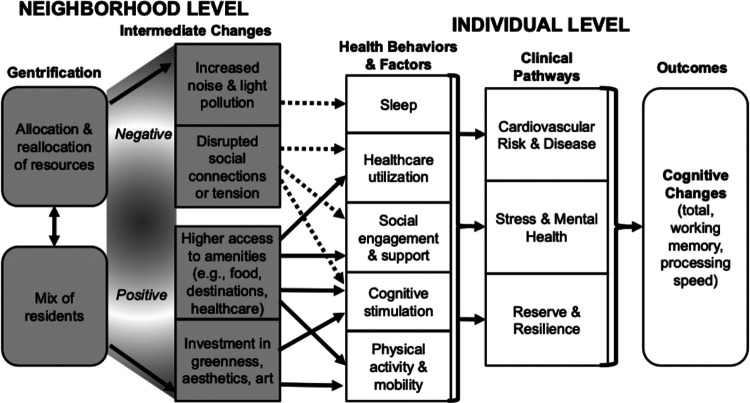
Conceptual model of gentrification mechanisms and cognitive change. Gentrification operates through intermediate neighborhood-level changes that may be negative (increased noise and light pollution; disrupted social connections or tension) and/or positive (higher access to amenities; investment in greenness, aesthetics, and art). These neighborhood changes influence individual-level health behaviors and factors including sleep, healthcare utilization, social engagement and support, cognitive stimulation, and physical activity and mobility. These behaviors affect three clinical pathways, including cardiovascular risk and disease, stress and mental health, and reserve and resilience, which collectively shape cognitive changes across domains of total cognition, working memory, and processing speed. Dashed lines indicate negative pathways; solid lines indicate positive pathways.

Gentrification is the transition of neighborhoods from a state of disinvestment to reinvestment, attracting wealthier and more educated inhabitants and improving socioeconomic conditions.^3^ While gentrification may lead to enhanced neighborhood resources, prior studies highlighted gentrification's effect on the deterioration of the overall neighborhood social environment, especially for low-income, Black older adults.^4,5^ However, the complex relation between gentrification and cognitive function in older adults remains underexplored (Fig. 1).

Our study examines the association between gentrification and cognitive decline in a diverse cohort of older adults residing in six U.S. metropolitan areas and evaluates difference by race/ethnicity.

## 2. Methods

### 2.1 Population

The sample consisted of participants from the Multi-Ethnic Study of Atherosclerosis (MESA), a study of 6,814 U.S. adults aged 45–84 years without clinical cardiovascular disease at enrollment.^6^ Participants were recruited between 2000 and 2002 from six study sites (Baltimore MD, Chicago IL, Forsyth County NC, Los Angeles CA, New York NY, and St. Paul MN). After initial examination, participants attended six additional follow-up examinations. Of 6,814 MESA participants, we excluded 3,204 who died before or without contributing follow-up cognitive data, lacked baseline or follow-up cognitive assessments, or had invalid cognitive scores. We further excluded 233 participants with dementia diagnosis or Alzheimer's medication use prior to cognitive testing, ungeocoded addresses, or missing covariate data. The final analytic sample was n = 1,731.

The study was approved by a study-wide IRB and all participants gave written informed consent.

### 2.2 Neighborhood Gentrification Exposure

We used a composite measure of gentrification between 2000–2010.^7^ This measure identifies 2010 census tracts eligible to gentrify (i.e. lower median household income in 2000) and then classifies these tracts based on shifts between 2000 and 2010 in affordability (i.e., higher rent and higher home value) and in demographic composition (i.e., percentage residents with college degree). This produces three categories: (1) continually advantaged (i.e. ineligible to gentrify, those that are in the top quartile of median household income relative to their metropolitan area); (2) continually disadvantaged (i.e., eligible to gentrify but did not gentrify, below a median increase in the distribution of change in education or housing for their area in 2000–2010); and (3) gentrified (i.e., above a median increase in the distribution of change in education or housing for their area in 2000–2010). MESA participant addresses for their residence in 2000 were linked with the gentrification measure using 2010 census tract boundaries.

### 2.3 Cognitive Outcome

Cognitive outcomes measured at three exams 2010–2024. We assessed total Global Cognitive Composite (GCC), an overall cognitive indicator which is an average if the standardized measures for each of the three cognitive measures,^8^ including the: Cognitive Abilities Screening Instrument (CASI), Digit Symbol Coding (DSC), and Digit Span (DS, forwards and backwards). Standardized measures were computed for each test at each exam by subtracting the 2010 sample mean from each participant’s score and dividing the result by the standard deviation of the sample in 2010.

### 2.4 Covariates

Except where noted, covariates were updated at each exam. Sociodemographic characteristics included gender, race/ethnicity, education, and birthplace assessed at Exam 1; time varying factors included age, language of cognitive test administration, total gross family income (modeled continuously using the midpoint of 15 categories), and current employment status.

Health factors included APOE-ε4 carrier status (≥ 1 allele)^9^ measured at Exam 1, hypertension,^10^ diabetes,^11^ cancer, body mass index (BMI), and prior cardiovascular disease events (myocardial infarction, and resuscitated cardiac arrest, angina pectoris, or stroke).

Behavioral and social factors included alcohol use, current cigarette smoking (current vs. former/never), moderate-to-vigorous physical activity (MET-minutes per week)^12,13^, depressive symptoms (CES-D), and perceived social cohesion.^14^

### 2.5 Analyses

We used linear mixed-effects models including random intercepts for census tract and participant to account for clustering and repeated measures, with an unstructured covariance matrix for within-person errors. To address potential bias due to loss to follow-up (LTF), all models used inverse probability weights (IPW)^15^ estimated from logistic regression models using baseline covariates. Separate models death and other LTF modeled separately; final stabilized weights were the product of inverse probabilities from each model, truncated at the 95th percentile.

#### Sensitivity Analyses

Sensitivity analyses included (1) modeling each GCC component test and (2) excluding participants who moved after 2000 (n = 956, 35%) to evaluate exposure misclassification.

## 3. Results

MESA participants living in continually disadvantaged neighborhoods were more likely to be older, Black, lower education and income, not employed, have higher BMI, and the APOE-ε4 allele compared to those living in neighborhoods that were continually advantaged or gentrified (Table 1). Compared with excluded participants, our analytic sample was younger, more likely to be White, higher socioeconomic status, and healthier at Exam 1 (see Supplemental Table 1).

Over 5 years of follow-up, global cognitive performance declined in all neighborhood groups. In age-adjusted models, the mean 5-year change (in standard deviation units) was − 0.16 (95% CI:−0.17, −0.14) for gentrified neighborhoods, − 0.14 (− 0.15, − 0.12) for continually disadvantaged, and − 0.12 (− 0.15, − 0.10) for continually advantaged neighborhoods (global p = 0.04). In fully adjusted models, patterns were similar but did not reach statistical significance (global p = 0.0604). Further adjustment for factors in presumed causal pathway did not alter the estimates (See Table 2). We found no evidence of effect modification by race/ethnicity.

Sensitivity analyses including models of the component cognitive functional measures were consistent with the results from our primary models, with strongest results observed for Digit Span tests representing working memory function. In the fully adjusted model, the mean 5-year change was − 0.63 (95% CI:−0.76, −0.50) for gentrified neighborhoods, for − 0.39 (95% CI:−0.51, −0.28) continually disadvantaged, and − 0.15 (95% CI:−0.31, 0.02) for continually advantaged (global p < 0.0001). Models which excluded participants who moved during follow-up were similar in magnitude to our overall results (See Supplemental Table 2).

## 4. Discussion

In this population of older adults, residing in a gentrifying neighborhood was associated with modestly steeper decline in global cognitive function compared to continually disadvantaged neighborhoods. While not statistically significant after adjustment for confounding factors, this pattern suggests a gradient in which neighborhood socioeconomic stability, rather than advantage per se, may shape late-life cognitive trajectories.

Despite a growing body of health research evaluating gentrification, none have quantified the role of gentrification on changes in cognition.^16^ One study of older adult health found that residents of gentrifying neighborhoods reported better overall health but worse mental health compared with those in consistently disadvantaged or consistently advantaged neighborhoods.^17^ This suggests complex and potentially competing mechanisms linking gentrification to health in later life. That study examined 40 years of neighborhood change, whereas we observed 10 years; our shorter period may have been insufficient to see significant differences in cognitive trajectories in our sample. A larger body of research consistently identified an association between neighborhood disadvantage and cognitive decline.^18^

Gentrification may bring economic growth and new infrastructure, and prior studies linked enhanced neighborhood amenities to increased physical activity and improved cardiometabolic risk factors.^19,20^ At the same time, these changes may erode social connectedness and support, particularly for residents who are “indirectly displaced” by changes in the neighborhood's character and social identity.^5^ Disruptions to social cohesion and stability may increase stress, social isolation, and poor sleep, factors associated with cognitive decline.^4^ Increasing cost of living in gentrifying neighborhoods increases financial strain, another stress-pathway linked to cognition, through depression, especially in minoritized communities.^21–24^

Our measure of gentrification, based on sociodemographic indicators, does not directly capture changes in the neighborhood cost of living and may not fully capture the complexity of neighborhood change or its relevance for cognitive health. Vulnerable groups, including renters, may experience gentrification as uniquely disruptive^4^ and those most affected may have relocated, potentially biasing associations towards the null. Future studies should directly assess older adults’ perceptions of neighborhood changes and their relation to cognitive trajectories^25^ to identify subgroups most likely to experience beneficial or adverse long term health cognitive effects. We did not directly evaluate environmental changes or perceived neighborhood change as mediators. Given that adjustment for potential behavioral, clinical, and social mediators (e.g., physical activity, cardiometabolic risk factors, social cohesion) did not meaningfully change the results, mechanisms remain unclear. Importantly, we used high-quality, longitudinal cognitive assessments sensitive to change within a well-characterized study population which allowed for careful confounder adjustment and development of inverse probability weights to account for potential selection bias due to loss to follow-up.

## 5. Conclusion

Overall, neighborhood gentrification in late life is unlikely to confer cognitive benefit and may be associated with modestly greater decline, particularly in memory function. Replication in larger samples is needed to confirm these patterns. Gentrification may introduce stressors related to displacement, social network disruption, or loss of place-based resources that are harmful for cognitive aging. Future research should examine these mechanisms directly.

## Supplementary Material

Supplementary Files

This is a list of supplementary files associated with this preprint. Click to download. y

- SupplementalMaterials.docx

## Figures and Tables

**Table 1:** Characteristics of Multi-Ethnic Study of Atherosclerosis (MESA) population stratified by gentrification status at Exam 5 (2010–2012, n = 1,731)

Sample characteristic	Continually Disadvantaged (Did not Gentrify) N = 735	Gentrified N = 633	Continually Advantaged (Not Eligible to Gentrify) N = 363	p-value^1^
**N (%) per group or mean (SD)**				
Age in years (mean [SD])	66.4 (8.0)	65.5 (8.1)	66.3 (7.5)	0.066
Male	416 (57%)	338 (53%)	175 (48%)	0.032
Race / Ethnicity				< .0001
White	230 (31%)	281 (44%)	241 (66%)	
Chinese American	65 (9%)	74 (12%)	54 (15%)	
Black	275 (37%)	122 (19%)	45 (12%)	
Hispanic	165 (22%)	156 (25%)	23 (6%)	
Language of administration				< .0001
English	614 (84%)	524 (83%)	341 (94%)	
Spanish	74 (10%)	61 (10%)	4 (1%)	
Chinese	47 (6%)	48 (8%)	18 (5%)	
Education				< .0001
Less than High School	83 (11%)	59 (9%)	3 (1%)	
Completed High School/some college	374 (51%)	316 (50%)	100 (28%)	
College degree	278 (38%)	258 (41%)	260 (72%)	
Total gross family income, $ (mean [SD])	$58,200 (39,000)	$59,000 (40,100)	$92,800 (44,300)	< .0001
Currently working full or part time	369 (50%)	353 (56%)	230 (63%)	0.0002
APOE-s4 allele	210 (29%)	145 (23%)	88 (24%)	0.046
Current Cigarette Smoker	333 (45%)	299 (47%)	154 (42%)	0.070
**N (%) per group or mean (SD)**				
Hypertension	406 (55%)	310 (49%)	161 (44%)	0.0016
Cardiovascular event	35 (5%)	26 (4%)	15 (4%)	0.811
Body Mass Index, kg/m^2^ (mean [SD])	29.4 (5.6)	28.7 (5.8)	27.4 (5.0)	< .0001
Moderate and vigorous physical activity, MET-Min/Week (mean [SD])	5,700 (6,040)	6,160 (8,050)	5,330 (4,950)	0.148
Perceived social cohesion^2^ (mean [SD])	3.7 (0.6)	3.7 (0.6)	3.9 (0.6)	< .0001
Moved during follow up	387 (53%)	345 (55%)	224 (62%)	0.016
Study Site				< .0001
Wake Forest	123 (17%)	61 (10%)	103 (28%)	
Columbia	140 (19%)	124 (20%)	30 (8%)	
Johns Hopkins	145 (20%)	63 (10%)	24 (7%)	
Minnesota	110 (15%)	195 (31%)	23 (6%)	
Northwestern	93 (13%)	72 (11%)	132 (36%)	
UCLA	124 (17%)	118 (19%)	51 (14%)	

1 p-value from chi-square test for categorical variables and one-way ANOVA models for continuous variables.

2 Ranges 1–5. Higher value indicates a more cohesive neighborhood.

**Table 2:** Associations between Gentrification Status (2000–2010) and Cognitive Status (2010–2024) from Linear Mixed Effects Models, Multi-Ethnic Study of Atherosclerosis (MESA), n = 1,731

Outcome		5-year change in outcome by gentrification status		
	Exposure	Age-adjusted^1^	Fully Adjusted^2^	Fully Adjusted + Potential Mediators^3^
		β per 5 years (95% CI)	β per 5 years (95% CI)	β per 5 years (95% CI)
Global Cognitive Score Decline				
	Gentrified	−0.16 (−0.17, −0.14)	−0.15 (−0.17, −0.14)	−0.15 (−0.17, −0.13)
	Continually Advantaged	−0.12 (−0.15, −0.10)	−0.12 (−0.14, −0.10)	−0.12 (−0.14, −0.09)
	Continually Disadvantaged	−0.14 (−0.15, −0.12)	−0.13 (−0.15, −0.12)	−0.13 (−0.14, −0.11)
	p-value^4^	0.0425	0.0604	0.0637

1 Model 1 = Gentrification+Years since Exam 5 + Gentrification*Years + Age+Age*Years; random intercepts for 2010 census tract and person (5-year change in outcome by gentrification status)

2 Model 2 = Gentrification+Years since Exam 5 + Gentrification*Years + Age+Age*Years+Gender+Race/Ethnicity+Language of administration+Education+Income+Working status+APOE4; random intercepts for 2010 census tract and person

3 Model 3 = Gentrification+ Years since Exam 5 + Gentrification*Years + Age+Age*Years+Gender+Race/Ethnicity+Language of administration+Education+Income+Working status+APOE4 + Current Smoking+Hypertension + CVD Event+Intentional exercise + BMI+Person-level perceived social cohesion; random intercepts for 2010 census tract and person

4 5-year change columns: p-value is the Type III test of the Gentrification × Years interaction, reflecting whether the rate of cognitive decline differs across gentrification groups overall. Estimates for each group represent group-specific rates of change (slopes) rather than contrasts against a referent; accordingly, no single reference group is designated for these columns. Pairwise contrasts between specific gentrification groups were not the primary focus of analysis.

## Data Availability

The data that support the findings of this study are derived from the Multi-Ethnic Study of Atherosclerosis (MESA) and are available through two public NIH repositories. Non-genetic MESA data are available through the National Heart, Lung, and Blood Institute's Biorepository (BioLINCC) at https://biolincc.nhlbi.nih.gov, and genetic data are available through the database of Genotypes and Phenotypes (dbGaP) at https://www.ncbi.nlm.nih.gov/gap. Data are also accessible to approved investigators through the MESA Research Databases via the MESA website at https://www.mesa-nhlbi.org. Access to MESA data requires completion of a data access application, institutional IRB approval, and execution of a MESA Data and Materials Distribution Agreement (DMDA). Data are released to NIH repositories three months prior to study completion or upon publication of findings, whichever comes first, and remain available indefinitely. Additional information about data access procedures is available at https://www.mesa-nhlbi.org.
